# Procedural justice, managerial leadership and gender-based harassment in the workplace: longitudinal cross-lagged associations in a Swedish cohort

**DOI:** 10.3389/fpsyg.2026.1783045

**Published:** 2026-03-25

**Authors:** Paraskevi Peristera, Johan Paulin, Emma Cedstrand, Gun Johansson, Anna Nyberg

**Affiliations:** 1 Department of Psychology, Stockholm University, Stockholm, Sweden; 2 Department of Occupational Health, Psychology, and Sports Sciences, University of Gävle, Gävle, Sweden; 3 Unit of Occupational Medicine, Institute for Environmental Medicine, Karolinska Institutet, Stockholm, Sweden; 4 Center of Occupational and Environmental Medicine, Stockholm Region, Stockholm, Sweden; 5 Department of Public Health and Caring Sciences, Uppsala University, Uppsala, Sweden

**Keywords:** longitudinal cohort study, managerial leadership, procedural justice, SEM analysis, workplace gender-based harassment

## Abstract

**Purpose:**

Gender-based harassment (GBH) is a persistent workplace problem with substantial consequences for employees and organizations. Although organizational conditions are assumed to shape harassment risk, longitudinal evidence on modifiable organizational antecedents remains limited. Building on organizational justice theory and the perpetrator–predation paradigm, this study examines whether procedural justice and supportive managerial leadership prospectively predict GBH.

**Methods:**

Using data from the Swedish Longitudinal Occupational Survey of Health (SLOSH) from 2018 and 2020 (*n* ≈ 5000), we estimated cross-lagged associations between self-reported procedural justice, managerial leadership, and experienced and witnessed GBH within a structural equation modelling framework.

**Results:**

Experienced GBH was reported by 3%–4% and witnessed GBH by 6%–9% of the working sample. Higher procedural justice in 2018 was associated with a very small decreased risk of experienced (*β* = −0.001, *p* = 0.018) and a small decreased risk of witnessed (*β* = −0.052, *p* < 0.001) GBH in 2020. Higher managerial leadership scores were associated with a small decreased risk of experienced GBH (*β* = −0.036, *p* = 0.017). No reverse associations were observed.

**Conclusion:**

The findings provide longitudinal evidence that organizational justice and leadership, although relatively weak, shape harassment risk environments over time. By demonstrating prospective associations while accounting for reverse and stability effects, the study advances understanding of organizational antecedents of GBH beyond predominantly cross-sectional research.

## 1 Introduction

Gender-based harassment (GBH) is one of the most prevalent forms of workplace mistreatment and constitutes a major work environment problem (Cortina and Areguin, 2021; Dresden et al., 2018; Leskinen and Cortina, 2014; Leskinen et al., 2011). According to the International Labour Organization (International Labour Conference, 2019) GBH refers to “violence and harassment directed at persons because of their sex or gender, or affecting persons of a particular sex or gender disproportionately, and includes sexual harassment.” It encompasses verbal, non-verbal, and physical acts, including derogatory remarks, intimidation, and exclusion of individuals who do not conform to stereotypical gender norms (Berdahl, 2007; Dionisi and Barling, 2018; Fitzgerald et al., 1995; Hershcovis and Barling, 2010; Leskinen and Cortina, 2014). GBH is associated with substantial health risks not only for targets (Blindow et al., 2021; Blindow et al., 2022; Jahnke et al., 2019; Mensah et al., 2022; Miner-Rubino and Cortina, 2007; Muhonen, 2016; Richman-Hirsch and Glomb, 2002) but also for witnesses, who may develop fear, anger, empathy distress, or concerns for their own safety (Gabriel et al., 2024; McMahon et al., 2017; Miner and Eischeid, 2012). Taken together, these findings indicate that GBH is not only an individual experience but also a broader organizational phenomenon, with implications for workplace climate, norms, and well-being.

A growing body of research suggests that GBH does not occur in isolation but is closely tied to organizational conditions. Workplace incivility is recognized as a reflection of the workplace climate or culture rather than an isolated individual phenomenon, and organizational conditions therefore play a central role in shaping the likelihood of incivility (Andersson and Pearson, 1999; Cortina et al., 2001; Han et al., 2022; Hershcovis, 2011). Han et al. (2022) proposed a model in which three factors interact to shape the likelihood of incivility and harassment. The first two categories describe dispositional and demographic characteristics of the individuals while the third category captures environmental conditions such as passive leadership, the absence of civility norms, and a climate of incivility. Organizational justice and managerial leadership are considered particularly influential in shaping norms that either prevent or enable the occurrence of incivility These organizational factors are especially important because they are modifiable and can be targeted through interventions (Harold and Holtz, 2015; Walsh et al., 2012). The “perpetrator–predation paradigm” proposed by Cortina et al. (2018) further emphasizes that perpetrators exploit environments that tolerate misconduct, pointing to the central role of organizational structures, ethical norms, and leadership in either constraining or enabling harassment. According to this framework, perpetrators are more likely to engage in incivility when organizations have high tolerance or lack clear accountability for such behaviors. Taken together, these perspectives converge on the notion that organizational conditions shape the likelihood of GBH by communicating acceptable behavior, accountability, and consequences, thereby influencing whether harassment is constrained or allowed to persist. Our argument centres on the behavior of perpetrators, who are more likely to exploit environments that tolerate misconduct, take advantage of vulnerabilities, and engage in incivility when they perceive minimal consequences for their actions. Managerial leadership and workplace culture, in which perceived justice is a key component, therefore play pivotal roles in shaping norms that either prevent or allow incivility and harassment to thrive.

Procedural justice is a subdimension of organizational justice and refers to the perceived fairness and transparency of decision-making processes (Colquitt et al., 2013). Low levels of procedural justice have been linked to poor health outcomes (Colquitt et al., 2013; Cropanzano and Greenberg, 1997; Eib et al., 2018; Elovainio et al., 2002; Persson et al., 2020) and a higher likelihood of workplace harassment (Bildt, 2005; Butler and Chung-Yan, 2011; Cogin and Fish, 2009; Lee, 2018; Park, 2021). Research also suggests that high procedural justice may buffer the negative impact of bullying on well-being (Hsu et al., 2019). In the present study, we build on this body of research by applying a longitudinal research design to further examine the role of procedural justice in the workplace, with a specific focus on gender-based harassment.

Managerial leadership is another crucial organizational condition that is associated with employee health (Montano et al., 2017). Supportive leadership behaviors, such as clear communication, feedback, and empowerment, have been found to be associated with improved employee health and reduced sickness absence (Nyberg et al., 2009; Schmidt et al., 2018; Schmidt et al., 2014; Stengård et al., 2021). Conversely, passive or destructive managerial leadership has been associated with elevated risks of harassment and negative health effects (Cogin and Fish, 2009; Lee, 2018; Montano et al., 2017; Muhonen, 2016; Sadler et al., 2017). Cross-sectional studies suggest that managerial leadership plays a central role in whether harassment is reported, tolerated, or prevented (Bergman et al., 2002; Bildt, 2005; Daniel et al., 2019). The concept of managerial leadership used in the present study evaluates the presence of an attentive and supportive leader through measures of feedback, support, clear goals, and empowerment. Whereas procedural justice reflects formal organizational conditions, managerial leadership refers to the relationship between manager and employee, in which organizational norms are enacted in everyday work practices.

Witnessing harassment is an important dimension of GBH. The number of employees who observe harassment may indicate how tolerant an organization is, as perceived tolerance often shapes attitudes and behavior more than formal policies. Thus, witnessing GBH can reflect a workplace culture that fails to address harassment effectively.

Taken together, previous research indicates that organizational factors such as procedural justice and managerial leadership are likely to shape the risk of GBH in the workplace and highlights the importance of studying environmental variables to target in interventions to protect employees from workplace GBH (Havaei, 2021; Lopes et al., 2015; Zhou et al., 2017). Although previous research has linked organizational justice and leadership to GBH, most existing studies are cross-sectional and therefore unable to disentangle temporal ordering or rule out reverse associations. As a result, it remains unclear whether procedural justice and managerial leadership function as antecedent organizational conditions shaping harassment risk, or whether they primarily reflect employees’ *post hoc* interpretations of workplaces in which harassment has already occurred. By applying a longitudinal cross-lagged design, the present study moves beyond correlational evidence and tests whether these organizational factors prospectively predict both experienced and witnessed GBH while accounting for stability and potential reverse effects. In doing so, the study refines organizational justice theory and the perpetrator–predation paradigm by conceptualizing procedural justice and managerial leadership not as strong individual-level predictors, but as contextual organizational conditions that may regulate harassment risk environments over time.

The overall aim of the present study was to examine associations between organizational conditions, such as procedural justice and managerial leadership, and GBH. Our specific aims were to estimate the cross-lagged associations between:

1. Procedural justice and experienced GBH;
2. Procedural justice and witnessed GBH;
3. Managerial leadership and experienced GBH;
4. Managerial leadership and witnessed GBH.

We hypothesize that higher levels of procedural justice and supportive leadership contribute to lower experienced and witnessed GBH over time and that there are no statistically significant reverse associations.

## 2 Materials and methods

### 2.1 Study sample and design

Data were drawn from the Swedish Longitudinal Occupational Survey of Health (SLOSH), which is approximately representative of the Swedish working population. SLOSH started in 2006 and includes participants of the Swedish Work Environment Survey (SWES) 2003. SWES participants, in turn, consist of a subsample of gainfully employed people aged 16–64 from the Labor Force Survey (LFS). In later SLOSH waves, additional SWES cohorts have been added, resulting in a sample size of over 40,000 individuals (Magnusson Hanson et al., 2018). The SLOSH questionnaires consist of detailed questions about working life, general life situation, health and wellbeing. The authors did not have access to information that could identify individual participants during/after data collection. In this study, we included participants who reported working 30% or more of full-time in both 2018 and 2020 (*n* = 4994), while we excluded those who were self-employed. For the analyses of the variable witnessed GBH, participants (*n* = 283) who reported having experienced GBH at work in the period 2018–2020 were excluded. The Regional Research Ethics Board in Stockholm approved the study (No: 2019-05590) as well as the SLOSH data 2018 and 2020 (No: 2017/2535-32; 2019-06331).

This study adopts a longitudinal observational design to examine temporal associations between organizational conditions and GBH. The analytical strategy is guided by a contextual and organizational lens, in which procedural justice and managerial leadership are conceptualized as modifiable workplace conditions that may shape the risk environment for GBH. Cross-lagged panel analyses are employed to assess both prospective and reverse associations over time, while accounting for the temporal stability of the studied constructs. This approach allows us to align the longitudinal design with the overall research objective of examining directional associations.

### 2.2 Measures

#### 2.2.1 Procedural justice

Procedural justice was measured using a 7-item index with responses given on a 5-point scale, ranging from 1 = “strongly agree” to 5 = “strongly disagree” (Moorman, 1991). The seven procedural justice items ask questions about whether decisions are taken on the basis of correct information; decisions are consistent; bad decisions can be revoked or changed; all sides affected by the decision are represented; everyone can give their opinion in matters of immediate personal concern; feedback is provided regarding the consequences of decisions and people are informed accordingly; and it is possible to obtain a more detailed account of the information that underlies decisions if needed (Leineweber et al., 2016). To measure procedural justice, we obtained a sum of the seven items using a reversed scale so as to higher values indicate more positive perceptions of procedural justice (Cronbach’s alpha = 0.72).

#### 2.2.2 Managerial leadership

Managerial leadership was measured with an index, based on nine items that rate the behavior of managers, with response scale from 1 = “yes, often” to 4 = “No, never.” These items constitute the managerial leadership dimension of the Stress Profile Questionnaire (Nyberg et al., 2009; Setterlind and Larsson, 1995; Stengård et al., 2021) and measures if the employee gets the information he needs from his manager; if the manager is good at pushing through and carrying out changes; if the manager explains in a clear way the expectations he has on the employee; if the manager explains goals and sub-goals; if the manager shows that he/she cares about the employee; if the employee feels sufficient power in relation to his responsibilities; if the manager take time to be involved in the employees’ professional development; if the manager encourages employee involvement in work scheduling; and if the employee is praised when doing something good. To get our index, we reversed the scores and calculated the sum of the above-mentioned items, creating a variable with values ranging from 1 to 36 where higher values indicate better ratings of managerial leadership (Cronbach’s alpha = 0.65).

#### 2.2.3 Experienced GBH

To measure GBH we use three separate SLOSH questions, indicating whether during the last 6 months, the respondent had been subject to harassment by managers, co-workers or others (e.g., customers, clients, patients or students) because of his/her gender. Due to the highly skewed distribution of responses, with a majority of participants reporting minimal or no exposure, we dichotomized the GBH variable for the primary analyses. More specifically, we created a binary variable, based on the various response alternatives (1 = “yes, one or more times during the week”; 2 = “yes, one or more times per month”; 3 = “yes, sometime during the last 6 months”; 4 = “no”), with 1 = “yes” indicating a positive answer (response options 1 to 3) and 0 = “no” indicating a negative answer (response option 4).

#### 2.2.4 Witnessed GBH

This was measured with a single question: “Have you in the last 6 months heard of or seen someone in your workplace be subjected to harassment based on gender?” Participants answered on a four-point Likert scale ranging from 1 = “yes, one or more times per week” to 4 = “no, never.” As for the experienced GBH variable responses were dichotomized with the value 1 = “yes,” and 0 = “no.”

#### 2.2.5 Covariates

We considered several demographic and job-related variables in our analyses that previous research has suggested as potential confounders (Blindow et al., 2021; Mensah et al., 2022). More specifically, we controlled for age, gender, education, marital status, income, country of birth and origin of parents, and type of occupation, which were all obtained by linkage of questionnaire data to registers. Age was a categorical variable, with the following age groups: 0 “<35” 1 “35–44” 2 “45–54” 3 “55–64” 4 “65+.” Sex was coded into ‘0’ for male and ‘1’ female; marital status was coded into “1” married/co-habiting respondents and “0” otherwise. The logarithm of the disposable income was considered. Education was categorized in 4 groups: 1 “up to 9 yrs. of education”; 2 “up to 12 yrs. of education”; 3 “university education<3 yrs”; 4 “university education >3 yrs.” Country of birth was dichotomized as “Swedish” and “Other.” Origin of parents was categorized as “One or both parents born in Sweden” and “Parents born outside Sweden.” Occupations were classified according to the Swedish Standard Classification of Occupations (SSYK 2012), based on the International Standard Classification of Occupations (ISCO-08): 1 “Legislators, senior officials, managers” 2 “Professionals” 3 “Technicians” 4 “Clerks” 5 “Service workers” 6 “Agricultural and fishery workers” 7 “Craft workers” 8 “Machine Operators” 9 “Elementary occupations.”

Work-related variables included job demands and decision authority. Job demands were measured by four items of the Swedish Demand Control Questionnaire (DCSQ) (Chungkham et al., 2013), namely “Do you have to work very fast?”, “Does your work often involve conflicting demands?,” “Do you have enough time to do everything?,” “Does you work demand too much effort?.” Participants responded using a four-point Likert scale ranging from 1″ yes, often” to 4″ no, almost never/ never.” The mean of the four items (reverse scale for the first three items) was then calculated, with higher values indicating higher job demands. In the analysis, a binary variable, coded into “High job demands” for values above the median split and “Low job demands” otherwise, was used. Decision authority was measured by the items “deciding what to do at work” and “deciding how to do your work” of the Swedish Demand Control Questionnaire (DCSQ), with response values ranging from “Yes, often”1: to 4: “No, hardly ever/never” were used. A combined measure was calculated with higher values indicating lower decision authority. A binary variable, dichotomised on the basis of the median, was used in the analysis with 1 “Low decision authority” and 0 “High decision authority.”

### 2.3 Analytical strategy

Differences in study variables between 2018 and 2020 were estimated with two-tailed *t*-test. In line with the overarching analytical approach described above, cross-lagged analyses based on structural equation modelling were used to examine reciprocal associations between the exposure variables (procedural justice and managerial leadership) and outcome variables (experienced and witnessed GBH) over time. This approach allows examination of reciprocal associations between variables over time by estimating multiple relationships simultaneously. While the variables were modeled as observed rather than latent, SEM provides a flexible framework that accounts for longitudinal dependencies and missing data, yielding a more comprehensive understanding of temporal dynamics (Burkholder and Harlow, 2003; Little et al., 2007).

The first set of analyses (Model 1) estimated cross-lagged associations between procedural justice and managerial leadership in 2018 (t-1) and experienced GBH (GBH-E) and witnessed GBH (GBH-W) in 2020 (t), as well as the reverse paths. For each exposure–outcome combination, we tested both autoregressive paths to capture the stability of each variable over time (i.e., the extent to which prior levels of a variable predict its own future levels), and bidirectional cross-lagged effects, representing potential reciprocal influences between the exposure and the outcome across time (i.e., whether changes in one variable predict subsequent changes in the other, and vice versa).

In Model 2, demographic covariates were added, and in Model 3 both demographic and work-related covariates were included. Interaction terms between sex and exposure variables were tested to assess potential gender differences.

Model fit was evaluated using multiple indicators, which is recommended for reliable assessment (Kline, 1998). Fit was considered acceptable if RMSEA ≤ 0.08, CFI ≥ 0.90, and SRMR ≤ 0.08 (Hu and Bentler, 1999). All analyses were run in Mplus 8.4 (Muthén and Muthén, 1998). Missing data were handled using Full Information Maximum Likelihood (FIML), which is the default in Mplus for SEM estimation. FIML uses all available data points under the assumption that data are missing at random (MAR), thereby minimizing bias and maximizing statistical power compared with listwise deletion. Chi-square difference tests were used to compare fit across the three models. Following previous research (Mensah et al., 2022), we do not report estimates for the covariates, as they are not central to the research questions. The results illustrated a very good fit for all the models, as indicated by the RMSEA, CFI, and SRMR values within recommended thresholds (Appendix Tables A1, A2). Comparisons of nested models indicated that Model 3 seemed to fit the data significantly better than Model 2 and Model 1 (Δχ^2^_GBH-ProceduralJustice_ = 178.279, *p* < 0.001; Δχ^2^_GBH-ManagerialLeadership_ = 157.532, *p* < 0.001; Δχ^2^_GBH-W-ProceduralJustice_ = 9.669, *p* < 0.001; Δχ^2^_GBH-ManagerialLeadership_ = 34.623, *p* < 0.001). Cross-lagged effect sizes were assessed according to proposed thresholds (Orth et al., 2024): 0.03 (small), 0.07 (medium), and 0.12 (large).

## 3 Results

### 3.1 Descriptive statistics

The descriptive statistics for the study variables in SLOSH 2018 and 2020 are presented in Table 1. Taken together, these descriptive results indicate that both experienced and witnessed GBH were relatively uncommon in the sample and declined slightly over time, while mean levels of procedural justice and managerial leadership increased modestly between 2018 and 2020. Experienced GBH was reported by 3%–4% and witnessed GBH by 6%–9% of the working sample. Our sample consisted mostly of women (60%), of age-groups between 55 and 64 years old (38% in 2018; 43% in 2020), and most of the participants were married (59%) and highly educated (55% in both 2018 and 2020). In 2018, 4% of participants reported having been subjected to gender-based harassment, a proportion that declined to 3% in 2020. Moreover, 9% of participants indicated that they had witnessed gender-based harassment in 2018, compared to 6% in 2020. Procedural justice varied from a mean value of 22 to 24 between 2018 and 2020 while managerial leadership changed from a mean value of 28 in 2018 to a value of 29 in 2020. All the differences were statistically significant.

**Table 1:** Distribution of study variables for total sample in SLOSH waves in 2018 and 2020 (*n* = 4994).

Variable	Year			2018	2020
Gender (*n*, %)		
Women	2,982 (59.71)	2,982 (59.71)
Men	2,012 (40.29)	2,012 (40.29)
Age (*n*, %)		
<34 years	207 (4.14)	108 (2.16)
35–44 years	878 (17.58)	747 (14.96)
45–54 years	1,877 (37.59)	1,676 (33.56)
55–64 years	1,919 (38.43)	2,146 (42.97)
>64 years	113 (2.26)	317 (6.35)
Education (*n*, %)		
≤9 years	152 (3.05)	149 (2.98)
≤12 years	1,709 (34.25)	1,694 (33.92)
University < 3 years	402 (8.06)	401 (8.03)
University ≥3 years	2,727 (54.65)	2,750 (55.07)
Marital status (*n*, %)		
Not married / cohabited	2,068 (41.41)	2,068 (41.41)
Married / cohabited	2,926 (58.59)	2,926 (58.59)
Income		
≤329,999	1,418 (28.39)	985 (19.72)
330,000–399,999	1,189 (23.81)	1,100 (22.03)
400,000–509,999	1,094 (21.91)	1,252 (25.07)
≥510,000	1,293 (25.89)	1,657 (33.18)
Type of occupation (*n*, %)		
Legislators, senior officials, managers	563 (11.49)	568 (11.55)
Professionals	1,981 (40.43)	2,003 (40.74)
Technicians	900 (18.37)	902 (18.34)
Clerks	348 (7.10)	358 (7.28)
Service workers	605 (12.35)	602 (12.24)
Agricultural and fishery workers	17 (0.35)	22 (0.45)
Craft workers	234 (4.78)	226 (4.60)
Machine operators	193 (3.94)	189 (3.84)
Elementary occupations	59 (1.20)	47 (0.96)
Country of Birth (*n*, %)		
Sweden	4,578 (91.67)	5,016 (94.02)
Outside of Sweden	416 (8.33)	319 (1.46)
Origin of parents (*n*, %)		
One or both parents born in Sweden	4,578 (91.67)	4,578 (91.67)
Parents born outside of Sweden	416 (8.33)	416 (8.33)
Experienced gender-based harassment (*n*, %)*		
Yes	206 (4.12)	127 (2.54)
No	4,788 (95.88)	4,867 (97.46)
Witnessed gender-based harassment (*n*, %)*		
Yes	428 (8.57)	313 (6.27)
No	4,566 (91.43)	4,631 (93.73)
Procedural justice (M, SD)*	22.17 (6.58)	23.57 (6.59)
Managerial Leadership (M, SD)*	28.29 (5.28)	28.80 (5.03)
Job demands (M, SD)	2.51 (0.54)	1.86 (0.69)
Decision Authority (M, SD)	1.88 (0.68)	1.86 (0.69)

*p < 0.05; **p < 0.01; ***p < 0.001; ns, non significant.

Paired two-tailed *t*-test of relationship between respondents’ in 2018 and 2020.

### 3.2 Cross-lagged panel effects

To assess the robustness of associations to potential confounding, we estimated three sequential models as described above: Model 1 included only the focal constructs (procedural justice, managerial leadership, and GBH); Model 2 additionally adjusted for sociodemographic characteristics (age, gender, education, income, country of birth, origin of parents, and marital status); and Model 3 further adjusted for work environment factors (occupation, job demands, job control). In the current text, we focus our interpretation on the fully adjusted Model 3, as it provides the most conservative estimates while controlling for key demographic and workplace confounders. However, comparison across models revealed that the focal associations remained substantively stable across specifications, with only modest attenuation in Model 3 relative to Models 1 and 2 (see Appendix Table A3). This stability indicates that the observed associations are not primarily driven by demographic composition or general work environment characteristics, but represent distinct relationships between organizational justice, leadership, and gender-based harassment.

**Figure 1:**
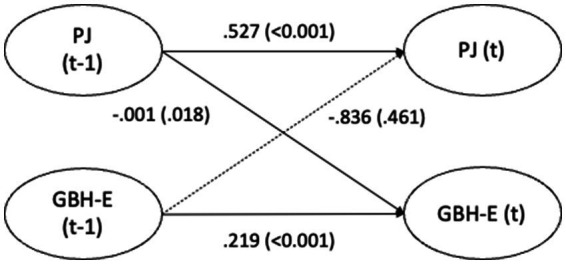
Paths between procedural justice (PJ) and experienced gender-based harassment (GBH-E) (*n* = 5,127) in SLOSH 2018 (*t*-1) and 2020 (*t*). Analyses adjusted for age, gender, education, income, country of birth, origin of parents, type of occupation, marital status, job demands, and job control. Statistically significant paths in solid lines.

**Figure 2:**
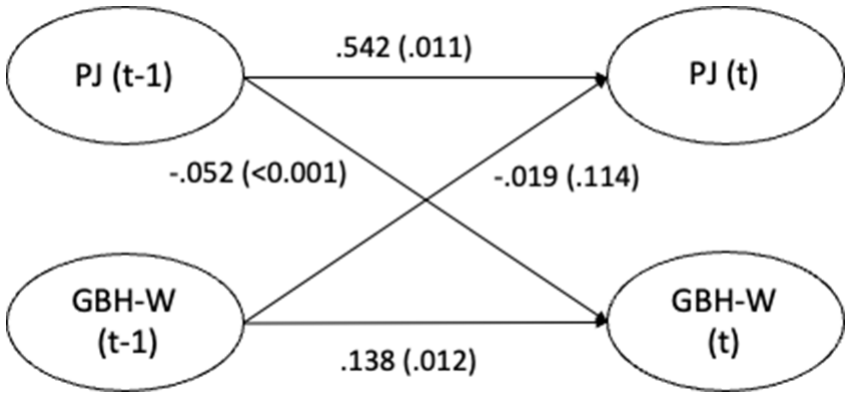
Paths between procedural justice (PJ) and witnessed gender-based harassment (GBH-W) (*n* = 4,871) in SLOSH 2018 (t-1) and 2020 (t). Analyses adjusted for age, gender, education, income, country of birth, origin of parents, type of occupation, marital status, job demands, and job control. Statistically significant paths in solid lines.

**Figure 3:**
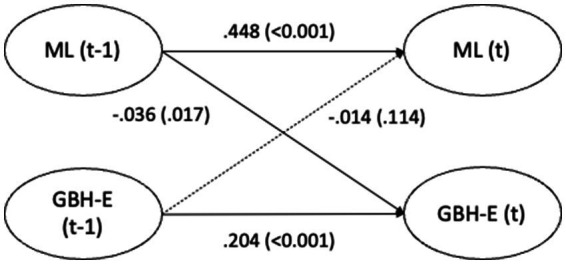
Paths between managerial leadership (ML) and experienced gender-based harassment (GBH-E) (*n* = 5,127) in SLOSH 2018 (t-1) and 2020 (t). Analyses adjusted for age, gender, education, income, country of birth, origin of parents, type of occupation, marital status, job demands, and job control. Statistically significant paths in solid lines.

**Figure 4:**
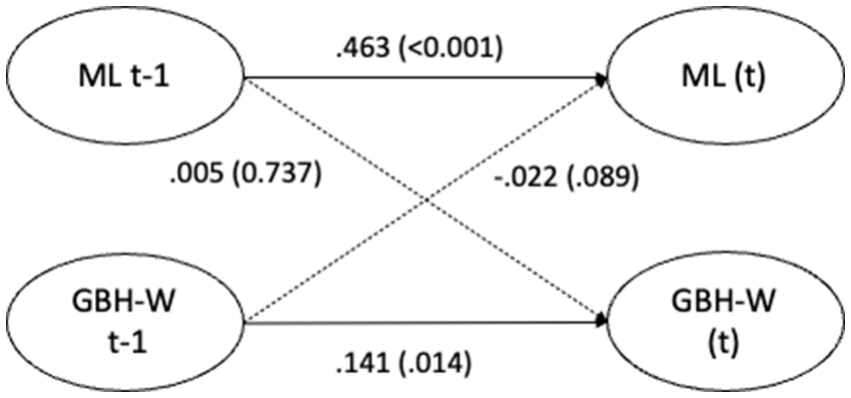
Paths between managerial leadership (ML) and witnessed gender-based harassment (GBH-W) (*n* = 4,871) in SLOSH 2018 (t-1) and 2020 (t). Analyses adjusted for age, gender, education, income, country of birth, origin of parents, type of occupation, demands, and job control. Statistically significant paths in solid lines.

The cross-lagged effects (standardized estimates) for Model 3 are presented in Figures 1–4 and in text below. Overall, the cross-lagged analyses revealed a pattern of small but consistent prospective associations between organizational conditions and subsequent reports of GBH, the exception being between managerial leadership and later witnessed harassment. Procedural justice and managerial leadership were more strongly associated with later reports of GBH than vice versa, while autoregressive paths for all constructs were substantial, indicating considerable stability over time. That the reverse associations from experienced or witnessed GBH to subsequent perceptions of procedural justice or managerial leadership were not statistically significant suggest that the observed longitudinal associations primarily run from organizational conditions to GBH, rather than the opposite direction.

#### 3.2.1 Associations between procedural justice and experienced GBH

Higher levels of procedural justice in 2018 were associated with slightly lower risk of GBH 2 years later (−0.001, *p* = 0.018). Although statistically significant, the magnitude of the association was very small and below conventional thresholds for small cross-lagged effects, and should therefore be interpreted as a marginal contextual shift rather than a substantively meaningful individual-level association. The reverse association, between experienced GBH in 2018 and procedural justice in 2020, was not statistically significant. Auto-regressive paths from 2018 to 2020 were stable for both procedural justice 0.527 (*p* < 0.001) and for experienced GBH 0.219 (*p* < 0.001).

#### 3.2.2 Associations between procedural justice and witnessed GBH

Higher levels of procedural justice in 2018 were associated with a small but statistically significant reduction in the risk of witnessing GBH in 2020. This association was stronger than the corresponding association for experienced GBH and remained robust after adjustment for covariates and autoregressive effects. Higher procedural justice in 2018 was associated with lower risk of witnessed GBH (small effect) at the workplace in 2020 (−0.052, *p <* 0.001). The reverse association was not statistically significant. Procedural justice in 2018 significantly predicted procedural justice in 2020 (0.542, *p <* 0.001) and witnessed GBH in 2018 was significantly associated with witnessed GBH 2020 (0.138, *p* = 0.012).

#### 3.2.3 Associations between managerial leadership and experienced GBH

Supportive managerial leadership 2018 significantly predicted experienced GBH in 2020, with a small effect size (−0.036, *p* = 0.017). The magnitude of this association was small, indicating that supportive managerial leadership was associated with a modest reduction in the risk of later experienced GBH. The reverse path between experienced GBH 2018 and managerial leadership 2020 was not statistically significant. The estimates for the auto-regressive paths for managerial leadership was 0.448 (*p* < 0.001) and for experienced GBH 0.204 (*p* < 0.001).

#### 3.2.4 Associations between managerial leadership and witnessed gender-based harassment

Supportive managerial leadership in 2018 significantly predicted managerial leadership in 2020 (0.463; *p* < 0.001) while witnessed GBH in 2018 significantly predicted witnessed GBH in 2020 (0.141; *p* = 0.014). The estimates for the paths between managerial leadership and witnessed GBH were not statistically significant, as shown in Figure 4. That no statistically significant cross-lagged associations were observed between managerial leadership and witnessed GBH suggests that while leadership may influence the risk of harassment for direct targets, witnessing GBH may reflect broader organizational dynamics that are less directly shaped by individual managerial behavior.

Taken together, the results point to a consistent pattern in which higher procedural justice and supportive managerial leadership are prospectively associated with a reduced risk of GBH over time, particularly with respect to witnessed GBH. Although effect sizes were small, the direction and consistency of the associations across outcomes and model specifications suggest that organizational conditions function as weak but systematic predictors of harassment risk in the workplace.

#### 3.2.5 Gender differences

Given that GBH disproportionately affects women, interaction analyses were conducted to examine whether the longitudinal associations between organizational conditions and GBH differed by gender. This was done by including an interaction term between gender and the exposure variables in Model 4. These analyses did not reveal any statistically significant gender differences, indicating that the observed associations operated in a similar manner for women and men.

## 4 Discussion

In this longitudinal study of Swedish workers, we found that higher levels of procedural justice predicted reduced risk of experiencing GBH (very small effect) and witnessing GBH (small effect) over time, while supportive managerial leadership predicted reduced risk of experiencing GBH (small effect) only. No reverse associations or gender differences were observed. These findings support theoretical models highlighting organizational conditions as determinants of workplace harassment (Cortina et al., 2018; Einarsen and Einarsen, 2021; Han et al., 2022).

Although the observed associations were in the expected direction, their magnitude was small. This pattern must be interpreted in light of the conservative analytical strategy employed. GBH is a relatively low-prevalence outcome, and its occurrence is shaped by a complex interplay of individual, relational, and organizational conditions. When cross-sectional associations and reverse paths are accounted for, as in the present cross-lagged models, effect estimates are likely to reflect incremental shifts in organizational risk environments rather than strong individual-level prediction.

### 4.1 Procedural justice and GBH

Previous studies have reported inconsistent associations between procedural justice and GBH with some cross-sectional studies identifying significant relationships while others report weak or non-significant associations. These inconsistencies may partly reflect differences in study design, as cross-sectional analyses are unable to disentangle temporal ordering or account for reverse associations. The present results extend earlier cross-sectional studies (Bildt, 2005; Butler and Chung-Yan, 2011; Cogin and Fish, 2009; Park, 2021) by showing that procedural justice predicts reduced GBH over time, even when adjusting for confounders and autoregressive effects. Importantly, the effect was evident not only for experienced GBH but also for witnessed GBH. Our findings support Han et al.’s (2022) model, which emphasizes that organizational rather than solely individual conditions are central to the risk of incivility and harassment. By showing that procedural justice predicts both experienced and witnessed GBH over time, this study provides longitudinal evidence that transparent and fair decision-making creates an organizational climate that protects not only direct targets but also bystanders. The results may also be interpreted in light of Cortina et al.’s (2018) perpetrator–predation paradigm, which argues that perpetrators exploit environments that tolerate misconduct. The finding that higher procedural justice decreases the likelihood of later GBH suggests that clear and just processes may lead to organizational intolerance for incivility, thereby reducing opportunities for harassment to occur. From this perspective, procedural justice can be viewed not merely as an indicator of organizational climate but also as a mechanism that constrains the organizational space in which harassment can thrive. The associations for both experienced and witnessed GBH are small, but this result should be interpreted with the understanding that the cross-sectional associations between justice and harassment are taken into account at both measurement points and that the time-lag between waves is rather long.

### 4.2 Managerial leadership and GBH

We also found that supportive managerial leadership was prospectively associated with reduced risk of experiencing GBH. This confirms prior research linking such leadership to lower harassment risks (Cogin and Fish, 2009; Lee, 2018; Muhonen, 2016; Sadler et al., 2017). However, the lack of association with witnessed GBH suggests that leadership may primarily affect the direct leader–employee relationship, whereas witnessing harassment reflects broader group dynamics less influenced by individual leadership behaviors (Grint, 2005; Yukl, 1989). In this context, witnessing GBH may be more associated to inter-relational factors on a personal or group level, such as shared norms or culture within the peer-group. These horizontal group dynamics, establishing what’s viewed as acceptable and normative, may better reflect what influences witnessing GBH than signals from the leadership. In contrast, procedural justice represents an organizational-level process, which may explain its broader reach to both direct targets and observers. This distinction aligns with theoretical perspectives emphasizing that procedural justice reflects organization-wide norms and decision-making structures, whereas managerial leadership represents a more proximal and relational mechanism. While justice perceptions may shape the general climate of tolerance for harassment, leadership practices may be particularly salient in situations involving direct interaction between managers and employees.

### 4.3 Reverse associations

We found no evidence that experiencing or witnessing GBH predicted later perceptions of procedural justice or managerial leadership. This is in contrast to a previous cross-sectional study showing positive associations between treatment by leaders and satisfaction after reporting GBH (Daniel et al., 2019). The absence of significant reverse associations (i.e., that GBH would affect later perceptions of justice or leadership) strengthens the argument that organizational conditions operate as causal antecedents rather than as mere correlates or consequences of harassment. This finding suggests that investments in procedural justice and supportive leadership may serve as genuinely preventive strategies, rather than solely reactive measures. At the same time, the absence of associations may also partly reflect selective attrition, as individuals exposed to more severe or repeated GBH may be underrepresented in longitudinal follow-up, for example due to health-related non-response or exit from employment.

### 4.4 Gender differences

The absence of gender differences in the prospective associations suggests that organizational conditions such as procedural justice and managerial leadership may operate in similar ways for women and men. This finding complicates assumptions that organizational antecedents of GBH necessarily function in gender-specific ways and instead points to the role of shared organizational climates and norms in shaping harassment risk across gender groups.

### 4.5 Contributions of the study

Our results underscore the importance of organizational conditions in preventing GBH from occurring in the workplace, for all employees, regardless of whether they are targets or witnesses of harassment. This distinction between experienced and witnessed GBH extends prior research that has primarily focused on direct targets and allows for a more nuanced understanding of how organizational conditions may shape both individual exposure and the broader workplace climate in which GBH occurs. These findings support theoretical models highlighting organizational conditions as central determinant of workplace harassment (Cortina et al., 2018; Einarsen and Einarsen, 2021; Han et al., 2022). The findings align with the concept of ethical infrastructure (Einarsen and Einarsen, 2021), which refers to the combination of formal systems (policies, procedures, reporting mechanisms) and informal systems (leadership behaviors, workplace culture) that work together to prevent unethical behaviors and promote respectful treatment. Strengthening procedural justice can be seen as a key element of the formal system of ethical infrastructure. Importantly, our longitudinal analyses go beyond prior cross-sectional evidence. By integrating organizational justice theory with the perpetrator–predation paradigm, the present findings furthermore extend existing theoretical perspectives on workplace harassment. Rather than positioning organizational conditions as strong causal drivers, the results suggest that procedural justice and managerial leadership weakly affect the risk for GBH. Even modest associations, when persistent over time, may contribute to cumulative differences in GBH across workplaces. From this perspective, the present findings help reconcile mixed results in the literature by demonstrating that organizational conditions may exert subtle, context-dependent influences that are best captured through longitudinal designs.

### 4.6 Strengths and limitations

A key strength of this study is its longitudinal design in a large, population-based cohort, which allowed us to examine prospective and reverse associations between organizational conditions and gender-based harassment within a conservative analytical framework. By simultaneously modelling autoregressive, cross-lagged, and reverse paths using structural equation modelling, we were able to account for temporal stability and bidirectionality, thereby strengthening inferences about the temporal ordering of associations. Another strength is the distinction between experienced and witnessed GBH (Bowes-Sperry and O’Leary-Kelly, 2005), which provides a more nuanced picture of how organizational factors shape both individual exposure and the broader workplace climate in which harassment occurs. At the same time, several limitations should be considered when interpreting the findings. First, all variables were self-reported, which may introduce common method variance. However, perceptions of procedural justice, leadership, and GBH are inherently subjective constructs, and self-report is therefore an appropriate method for capturing employees’ experiences of organizational conditions and mistreatment. Second, witnessed GBH was measured using a single item and both GBH outcomes were operationalized as binary variables. While this approach is common in large-scale surveys and allows for the inclusion of relatively rare events, it may have reduced variability and attenuated effect estimates. The use of more detailed, multi-item measures and continuous scaling in future studies may provide a more sensitive assessment of harassment experiences. Third, the two-year time lag between measurement waves may not fully capture shorter-term dynamics between organizational conditions and GBH. Changes in leadership practices or justice perceptions may influence harassment risk over shorter time frames, while longer-term effects may also unfold beyond the interval studied here. Thus, the estimated associations should be interpreted as reflecting average effects over a relatively long time span rather than immediate responses. Additionally, we chose to model procedural justice and managerial leadership as observed composite variables rather than latent constructs in order to ensure model parsimony and convergence stability in the SEM models. However, this approach does not account for measurement error, which is known to attenuate effect sizes and therefore the true underlying associations may be somewhat stronger than reported.

Another limitation relates to potential differential attrition across demographic groups. In the present sample, younger participants and individuals with migration background were less represented in the follow-up wave, resulting in an overrepresentation of older and Swedish-born participants over time. Previous research suggests that GBH may be more prevalent among younger workers and among individuals with migration background, and that these groups may also report less favourable psychosocial working conditions. If individuals belonging to higher-risk groups are underrepresented in longitudinal follow-up, this may lead to an underestimation of both GBH prevalence and associations between organizational conditions and GBH. Such patterns of differential survey participation are well documented in occupational cohort studies and should be considered when interpreting the generalizability and magnitude of the observed associations. Although the observed associations between organizational conditions and GBH were small in magnitude, these effect sizes are consistent with prior meta-analyses of workplace harassment predictors, which report small to medium sized correlations typically in the 0.06 to 0.10 range (Cortina and Berdahl, 2008; Willness et al., 2007). The small effect sizes observed in the present study may partly reflect the conservative design and modelling strategy employed. Despite the conservative modelling approach, the direction of associations was consistent across outcomes. Furthermore, even modest reductions in risk may translate into meaningful decreases in the number of harassment incidents across the workforce. Taken together, the strengths and limitations of this study highlight the value of longitudinal, population-based designs for advancing understanding of organizational antecedents of GBH.

## 5 Practical implications and directions for future research

Procedural justice and managerial leadership encompass organizational practices related to fair and consistent decision-making, clarity of expectations, and feedback aligned with those expectations. In line with organizational justice theory and the perpetrator–predation paradigm, such practices can be understood as organizational conditions that shape GBH over time. From a practical perspective, this highlights the importance of building an ethical infrastructure (Einarsen et al., 2017) by implementing a coordinated set of formal and informal measures. The formal measures could be policies, recurrent communications, formal surveillance, and sanctions. These formal components are most effective when introduced as an integrated system that explicitly frames GBH and other forms of harassment as unacceptable. Equally important are informal systems, such as cultivating a strong conflict-management culture and ensuring that managers model zero tolerance for harassment in their everyday interactions, attitudes, and responses. By reinforcing both formal structures and informal cultural norms, organisations can create an environment where employees feel safer, more empowered to report incidents, and more confident that GBH and other forms of harassment will be taken seriously and addressed effectively.

While the present findings suggest that procedural justice and managerial leadership have weak effects on GBH, important theoretical and empirical gaps remain. Future research should further examine how justice-related conditions are produced, reinforced, and interpreted within organizations, and how they interact with other organizational conditions such as formal policies, reporting systems, peer norms, and power structures. More fine-grained longitudinal designs, validated multi-item measures of GBH, and mixed-methods approaches incorporating perspectives of employees, witnesses, managers, and HR professionals may provide deeper insight into the mechanisms through which organizational conditions shape GBH across different contexts and groups.

## 6 Conclusion

This study was motivated by the limited longitudinal evidence on organizational antecedents of GBH and the need to move beyond predominantly cross-sectional findings in the field. By applying a conservative cross-lagged analytical framework in a large, population-based cohort, the study addressed an important gap in understanding how organizational conditions relate to both experienced and witnessed harassment over time.

The findings indicate that procedural justice and managerial leadership are prospectively associated with GBH, although the observed effects were small in magnitude. Rather than undermining their relevance, these modest associations highlight the importance of conceptualizing organizational conditions as contextual risk regulators rather than direct causal drivers of GBH. From a theoretical standpoint, the results refine organizational justice theory and the perpetrator–predation paradigm by demonstrating how organizational conditions may exert weak but systematic influences on GBH.

At the same time, the study’s limitations, including self-reported measures, binary operationalizations of GBH, and a two-year time lag, underscore the need for caution in interpretation and point to important directions for future research. More fine-grained longitudinal designs, improved measurement strategies, and integrative approaches combining quantitative and qualitative methods are needed to further unpack the mechanisms through which organizational conditions shape GBH.

## Data Availability

The datasets presented in this article are not readily available because given restrictions from the ethical review board and considering that sensitive personal data are involved, the data that support the findings of this study are not possible to make freely available. Access to the data may be provided to other researchers in line with Swedish law and after consultation with the Stockholm University legal department. Requests to access the datasets should be directed to data@slosh.se.
