# Supplementary material for: Procedural justice, managerial leadership and gender-based harassment in the workplace: longitudinal cross-lagged associations in a Swedish cohort
Source: Front Psychol. 2026 Mar 25;17:1783045. doi: 10.3389/fpsyg.2026.1783045 (PMC13056609; doi:10.3389/fpsyg.2026.1783045)
Supplement: Supplementary file 1 [file Table_1.docx]

Table A1: Fit Statistics for all models in the relationship between experienced gender-based-harassment (GBH-E), Procedural Justice and Managerial Leadership

| Model | | χ^2^ | df | RMSEA | CFI | SRMR | AIC | BIC | Comparison | Δχ^2^ | Δdf |  |
| --- | --- | --- | --- | --- | --- | --- | --- | --- | --- | --- | --- | --- |
|  | GBH-E/Procedural Justice | | | | | | | |  |  |  |  |
| Model 1 | | 872.586 | 5 | 0.001 | 0.999 | 0.001 | 14140.044 | 14192.449 |  |  |  |  |
| Model 2 | | 1565.584 | 13 | 0.001 | 0.999 | 0.001 | 26488.401 | 26377.643 | M2-M1 | 692.998*** | 8 |  |
| Model 3 | | 1743.863 | 19 | 0.001 | 0.999 | 0.001 | 25921.089 | 25997.444 |  | 178.279*** | 6 |  |
|  | GBH-E/Managerial Leadership | | | | | | | | M3-M2 |  |  |  |
| Model 1 | | 780.214 | 5 | 0.001 | 0.999 | 0.001 | -3001.161 | -2948.755 |  |  |  |  |
| Model 2 | | 1214.997 | 13 | 0.001 | 0.999 | 0.001 | 24329.552 | 24218.794 | M2-M1 | 434.783*** | 8 |  |
| Model 3 | | 1372.529 | 19 | 0.001 | 0.999 | 0.001 | 23753.386 | 23902.826 | M3-M2 | 157.532*** | 6 |  |

Significance level: ∗ ∗ ∗p < 0.001, ∗∗ < 0.05, ∗ < 0.10; n.s: non-significant.

Table A2: Fit Statistics for all models in the relationship between witnessed gender-based-harassment (GBH-W), Procedural Justice and Managerial Leadership

| Model | | χ^2^ | df | RMSEA | CFI | SRMR | AIC | BIC | Comparison | Δχ^2^ | Δdf |  |  |  |  |
| --- | --- | --- | --- | --- | --- | --- | --- | --- | --- | --- | --- | --- | --- | --- | --- |
|  | GBH-W/Procedural Justice | | | | | | | |  |  |  |  |  |  |  |
| Model 1 | | 1894.855 | 5 | 0.001 | 0.999 | 0.001 | 14140.044 | 14192.449 |  |  | 8 |  |  |  |  |
| Model 2 | | 1912.908 | 13 | 0.001 | 0.999 | 0.001 | 26488.401 | 26377.643 | M2-M1 | 18.053** |  |  |  |  |  |
| Model 3 | | 1922.577 | 17 | 0.001 | 0.999 | 0.001 | 25921.089 | 25997.444 | M3-M2 | 9.669** | 4 |  |  |  |  |
|  | GBH-W/Managerial Leadership | | | | | | | |  |  |  |  |  |  |  |
| Model 1 | | 1345.209 | 5 | 0.001 | 0.999 | 0.001 | -3001.161 | -2948.755 |  |  |  |  |  |  |  |
| Model 2 | | 1358.949 | 13 | 0.001 | 0.999 | 0.001 | 24329.552 | 24218.794 | M2-M1 | 13.74n.s. | 8 |  |  |  |  |
| Model 3 | | 1379.832 | 17 | 0.001 | 0.999 | 0.001 | 23753.386 | 23902.826 | M3-M2 | 20.883** | 4 |  |  |  |  |
|  | |  |  |  |  |  |  |  | M3-M1 | 34.623** | 12 |  |  |  |  |

Significance level: ∗ ∗ ∗p < 0.001, ∗∗ < 0.05, ∗ < 0.10; n.s: non-significant.

Table A3. Standardized estimates and p-values from SEM models 1, 2, and 3.

|  |  | Model 1 |  | Model 2 |  | Model 3 |  |
| --- | --- | --- | --- | --- | --- | --- | --- |
| From | To | β | p-value | β | p-value | β | p-value |
| PJ (t-1) | PJ (t) | 0.484 | <0.001 | 0.524 | <0.001 | 0.527 | <0.001 |
| GBH-E (t-1) | GBH-E (t) | 0.265 | <0.001 | 0.275 | <0.001 | 0.219 | <0.001 |
| PJ (t-1) | GBH-E (t) | -0.041 | 0.034 | -0.026 | 0.076 | -0.01 | 0.018 |
| GBH-E (t-1) | PJ (t) | -0.035 | 0.046 | -0.025 | 0.070 | -0.836 | (0.461 |
|  |  |  |  |  |  |  |  |
| PJ (t-1) | PJ (t) | 0.086 | <0.001 | 0.556 | <0.001 | 0.542 | 0.011 |
| GBH-W (t-1) | GBH-W (t) | 0.578 | <0.001 | 0.139 | <0.001 | 0.138 | 0.012 |
| PJ (t-1) | GBH-W (t) | -0.009 | <0.001 | -0.060 | <0.001 | -0.052 | <0.001 |
| GBH-W (t-1) | PJ (t) | -0.085 | 0.090 | -0.021 | 0.078 | -0.019 | 0.114 |
|  |  |  |  |  |  |  |  |
| ML (t-1) | ML (t) | 0.243 | <0.001 | 0.483 | <0.001 | 0.204 | <0.001 |
| GBH-E (t-1) | GBH-E (t) | 0.233 | <0.001 | 0.277 | <0.001 | 0.448 | <0.001 |
| ML (t-1) | GBH-E (t) | 0.098 | <0.001 | -0.054 | <0.001 | -0.036 | 0.017 |
| GBH-E (t-1) | ML (t) | 0.139 | <0.001 | -0.020 | 0.144 | -0.014 | 0.114 |
|  |  |  |  |  |  |  |  |
| ML (t-1) | ML (t) | 0.458 | <0.001 | 0.477 | <0.001 | 0.463 | <0.001 |
| GBH-W (t-1) | GBH-W (t) | 0.123 | <0.001 | 0.143 | <0.001 | 0.141 | 0.014 |
| ML (t-1) | GBH-W (t) | 0.000 | 0.711 | -0.006 | 0.700 | 0.005 | 0.737 |
| GBH-W (t-1) | ML (t) | -0.505 | 0.056 | -0.025 | 0.053 | -0.022 | 0.089 |

Model 1 Main variables only; Model 2: Model 1+ sociodemographic variables; Model 3: Model 2 + work-related variables; PJ Procedural Justice; ML Managerial Leadership; GBH-E Experienced Gender-Based Harassment; GBH-W Witnessed Gender-Based Harassment.
